# Clinical Value of ChatGPT for Epilepsy Presurgical Decision-Making: Systematic Evaluation of Seizure Semiology Interpretation

**DOI:** 10.2196/69173

**Published:** 2025-05-12

**Authors:** Yaxi Luo, Meng Jiao, Neel Fotedar, Jun-En Ding, Ioannis Karakis, Vikram R Rao, Melissa Asmar, Xiaochen Xian, Orwa Aboud, Yuxin Wen, Jack J Lin, Fang-Ming Hung, Hai Sun, Felix Rosenow, Feng Liu

**Affiliations:** 1 Department of Computer Science Schaefer School of Engineering & Science Stevens Institute of Technology Hoboken, NJ United States; 2 Department of Systems and Enterprises Schaefer School of Engineering & Science Stevens Institute of Technology Hoboken, NJ United States; 3 School of Medicine Case Western Reserve University Cleveland, OH United States; 4 Department of Neurology University Hospitals Cleveland Medical Center Cleveland, OH United States; 5 Department of Neurology School of Medicine Emory University Atlanta, GA United States; 6 Department of Neurology School of Medicine University of Crete Heraklion Greece; 7 Department of Neurology and Weill Institute for Neurosciences University of California, San Francisco San Francisco, CA United States; 8 Department of Neurology University of California, Davis Davis, CA United States; 9 H. Milton Stewart School of Industrial and Systems Engineering Georgia Institute of Technology Atlanta, GA United States; 10 Department of Neurology and Neurological Surgery University of California, Davis Davis, CA United States; 11 Fowler School of Engineering Chapman University Orange, CA United States; 12 Center of Artificial Intelligence Far Eastern Memorial Hospital New Taipei City Taiwan; 13 Surgical Trauma Intensive Care Unit Far Eastern Memorial Hospital New Taipei City Taiwan; 14 Department of Neurosurgery Rutgers Robert Wood Johnson Medical School Rutgers, The State University of New Jersey New Brunswick, NJ United States; 15 Department of Neurology Epilepsy Center Frankfurt Rhine-Main Goethe University Frankfurt Frankfurt am Main Germany; 16 Semcer Center for Healthcare Innovation Stevens Institute of Technology Hoboken, NJ United States

**Keywords:** seizure semiology, epileptogenic zone localization, ChatGPT, clinical value, large language models, AI for health care, epilepsy presurgical evaluation

## Abstract

**Background:**

For patients with drug-resistant focal epilepsy, surgical resection of the epileptogenic zone (EZ) is an effective treatment to control seizures. Accurate localization of the EZ is crucial and is typically achieved through comprehensive presurgical approaches such as seizure semiology interpretation, electroencephalography (EEG), magnetic resonance imaging (MRI), and intracranial EEG (iEEG). However, interpreting seizure semiology is challenging because it heavily relies on expert knowledge. The semiologies are often inconsistent and incoherent, leading to variability and potential limitations in presurgical evaluation. To overcome these challenges, advanced technologies like large language models (LLMs)—with ChatGPT being a notable example—offer valuable tools for analyzing complex textual information, making them well-suited to interpret detailed seizure semiology descriptions and accurately localize the EZ.

**Objective:**

This study evaluates the clinical value of ChatGPT for interpreting seizure semiology to localize EZs in presurgical assessments for patients with focal epilepsy and compares its performance with that of epileptologists.

**Methods:**

We compiled 2 data cohorts: a publicly sourced cohort of 852 semiology-EZ pairs from 193 peer-reviewed journal publications and a private cohort of 184 semiology-EZ pairs collected from Far Eastern Memorial Hospital (FEMH) in Taiwan. ChatGPT was evaluated to predict the most likely EZ locations using 2 prompt methods: zero-shot prompting (ZSP) and few-shot prompting (FSP). To compare the performance of ChatGPT, 8 epileptologists were recruited to participate in an online survey to interpret 100 randomly selected semiology records. The responses from ChatGPT and epileptologists were compared using 3 metrics: regional sensitivity (RSens), weighted sensitivity (WSens), and net positive inference rate (NPIR).

**Results:**

In the publicly sourced cohort, ChatGPT demonstrated high RSens reliability, achieving 80% to 90% for the frontal and temporal lobes; 20% to 40% for the parietal lobe, occipital lobe, and insular cortex; and only 3% for the cingulate cortex. The WSens, which accounts for biased data distribution, consistently exceeded 67%, while the mean NPIR remained around 0. These evaluation results based on the private FEMH cohort are consistent with those from the publicly sourced cohort. A group *t* test with 1000 bootstrap samples revealed that ChatGPT-4 significantly outperformed epileptologists in RSens for the most frequently implicated EZs, such as the frontal and temporal lobes (*P*<.001). Additionally, ChatGPT-4 demonstrated superior overall performance in WSens (*P*<.001). However, no significant differences were observed between ChatGPT and the epileptologists in NPIR, highlighting comparable performance in this metric.

**Conclusions:**

ChatGPT demonstrated clinical value as a tool to assist decision-making during epilepsy preoperative workups. With ongoing advancements in LLMs, their reliability and accuracy are anticipated to improve.

## Introduction

Epilepsy is one of the most common neurological diseases, affecting more than 70 million people worldwide [1], with approximately 50.4 per 100,000 people developing new-onset epilepsy each year [2,3]. For patients with drug-resistant focal epilepsy, surgical resection of the epileptogenic zone (EZ) provides an effective intervention to control seizure attacks.

The EZ is a theoretical definition given by Lüders et al [4], and its removal will make the patient seizure-free; thus, it can only be derived or validated after surgical ablation. Seizure semiology, referring to signs and symptoms exhibited and experienced by a patient during epileptic seizures, yields valuable clues on localizing the EZs [5,6]. In addition to semiology, presurgical evaluation involves multimodal brain imaging tools such as electroencephalography, stereo-electroencephalography, magnetoencephalography, magnetic resonance imaging, and functional magnetic resonance imaging [4,7]. To determine the ground truth of EZs, the postsurgical outcome information (whether or not the patient achieved seizure freedom) is used to validate the resected brain regions, with seizure-free status determined according to the International League Against Epilepsy criteria “Class I: Completely seizure-free; no auras” [8] or the following classification by Engel et al [9]: “Class I: Seizure free or no more than a few early, non-disabling seizures.”

Interpreting seizure semiology is a nontrivial task because it relies heavily on expert knowledge with inconsistent and incoherent descriptions, leading to variability and potential limitations in presurgical evaluation. A recent study used the conditional inference tree algorithm to interpret seizure semiology but achieved a maximum accuracy of only 56.1% across 5 ictal onset regions [10]. Advanced technologies such as large language models (LLMs) have emerged as potential solutions to address the challenges associated with interpreting semiology for the localization of EZs. LLMs, particularly ChatGPT, have demonstrated remarkable capabilities across a wide range of natural language processing tasks, including processing complex textual descriptions. Given the descriptive nature of seizure semiology, LLMs are well-positioned to address this challenge.

ChatGPT, developed by OpenAI [11], has exhibited exceptional capability at processing and interpreting extensive textual data by training on extensive textual data sets using supervised learning and reinforcement learning from human feedback, making it a promising tool for clinical applications, such as information retrieval, clinical decision support, and medical report generation [12-14]. Notably, a study conducted in February 2023 reported that ChatGPT successfully passed the United States Medical Licensing Examination [15,16], confirming its potential as a reliable source of medical information. The increasing application of ChatGPT for diagnosing various diseases inspired us to use it to interpret seizure semiology and to localize the EZs, potentially to be used as an artificial intelligence copilot for the presurgical evaluation of patients with epilepsy [16,17].

It is desirable to assess the clinical value of LLMs, such as ChatGPT, for interpreting semiology to localize EZ. Given that EZs can be categorized into 6 distinct brain regions, it is important to explore whether ChatGPT demonstrates varied precision at localizing these zones. We used the Laboratory for Computational Neuroimaging Cortical Lobes Dataset (LCN-CortLobes) classification system (FreeSurferWiki) [18] to provide a standardized anatomical framework to categorize EZs into 6 regions: frontal lobe, temporal lobe, parietal lobe, occipital lobe, cingulate cortex, and insular cortex. If ChatGPT achieved superior performance, particularly in specific brain regions or consistently across all regions, it would showcase its potential to assist with epilepsy presurgical decision-making.

## Methods

### Ethical Considerations

The institutional review board at Stevens Institute of Technology exempted the approval of this secondary data analysis study under protocol 2024-039 (N). All the extracted data were de-identified.

### Public and Private Data Cohorts

The performance of ChatGPT was first evaluated using a seizure semiology database compiled from publicly available studies in published peer-reviewed journals. Furthermore, to mitigate the risk of testing the performance with data that may have been used during ChatGPT’s training, another private data cohort was constructed based on electronic health records (EHRs) from Far Eastern Memorial Hospital (FEMH) in Taiwan for external validation.

The public data cohort was compiled from peer-reviewed research articles published in the past 20 years identified through a systematic search in PubMed using keywords including “seizure,” “seizures semiology,” “epilepsy,” and “epileptogenic zones” [19]. Each research paper includes a combination of individual and group case reports and details on the EZs. Relevant information, such as seizure semiology and patient-specific details (eg, age, gender, and handedness), was extracted from the text or tables within the publications. All the extracted cases have descriptions of both seizure semiology and validated EZs. The authors validated the EZ with good surgical outcomes [20]. In cases where multiple EZs were identified, multiple general regions were assigned accordingly.

**Figure 1:**
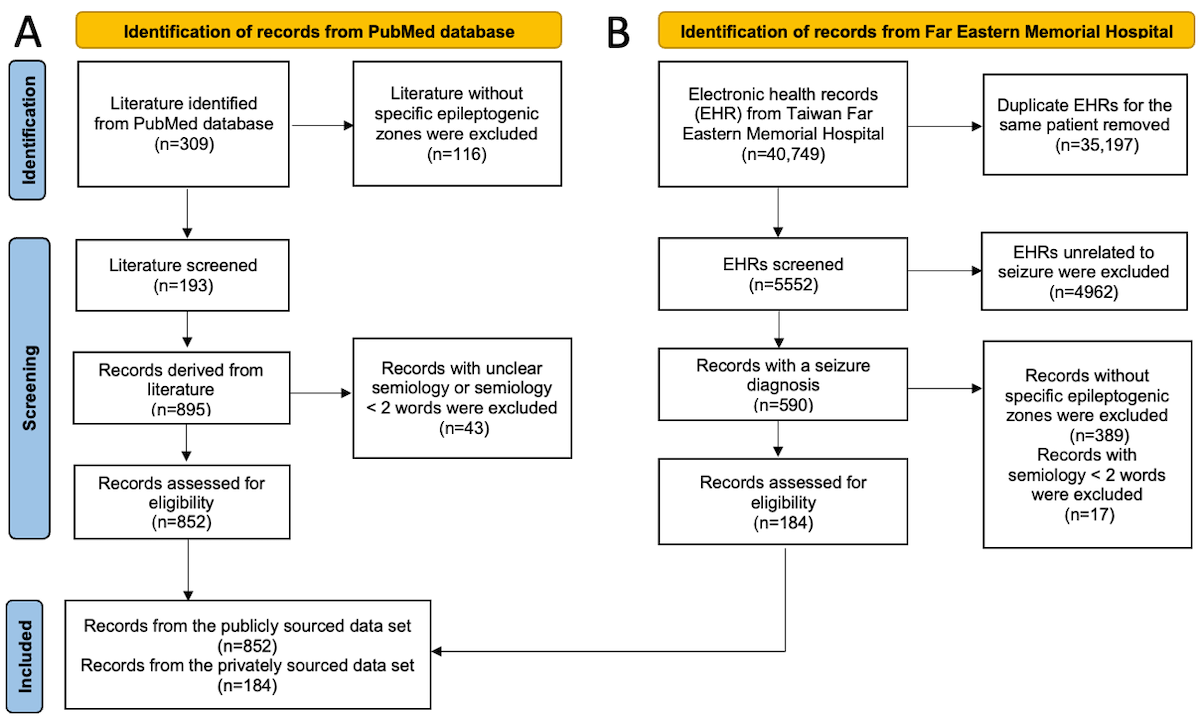
PRISMA (Preferred Reporting Items for Systematic Reviews and Meta-Analyses) flow diagram to construct (A) the publicly sourced and (B) the privately sourced data cohorts.

As illustrated in Figure 1A, the public data cohort initially included 309 publications. Among these, 116 studies were excluded due to presenting uncertain EZs, such as those specifying only hemisphere-level EZs (eg, “right hemispherectomy” or “left subtotal hemispherectomy”). From the remaining 193 studies, 893 cases were extracted with descriptions of seizure semiology, with an additional 43 being excluded due to unclear or nonspecific semiology descriptions, such as vague terms (eg, “non-specific aura”), descriptions shorter than 2 words, or aggregated data from large patient cohorts. The resulting database comprised 852 cases with detailed semiology descriptions and validated EZs. Examples of minimal semiology-EZ pairs include “speech arrest, lower of speech intensity – Insular Cortex (INS)” and “Aura with fear, aura with déjà vu – Cingulate Cortex (CING).”

The private data cohort was compiled from EHR at FEMH in Taiwan, covering 2017 to 2021. These EHRs included patients’ demographic information (eg, age, gender), clinical details (eg, diagnosis IDs and dates), symptoms, laboratory results, and clinicians’ diagnoses. Semiology was extracted from clinicians’ notes documenting patients’ symptoms, such as “dizziness and gait disturbance since late August 2016, no dysarthria or fever.” EZs were determined by integrating laboratory results with the clinical diagnoses validated by epileptologists.

As illustrated in Figure 1B, the initial EHR data set comprised 40,749 records, including redundant patient follow-up visits. After retaining the most recent record for each patient, the data set was reduced to 5552 cases. Further exclusions were made for records unrelated to epilepsy or diseases outside the scope of this study, resulting in 590 relevant records. Additional refinement involved the removal of cases with unclear semiology or undetermined EZs. Ultimately, the final data set consisted of 184 validated semiology-EZ pairs.

Conclusively, we evaluated ChatGPT’s performance using 2 data sets: a publicly sourced data set comprising 852 semiology-EZ pairs and a private FEMH data set containing 184 semiology-EZ pairs.

**Figure 2:**
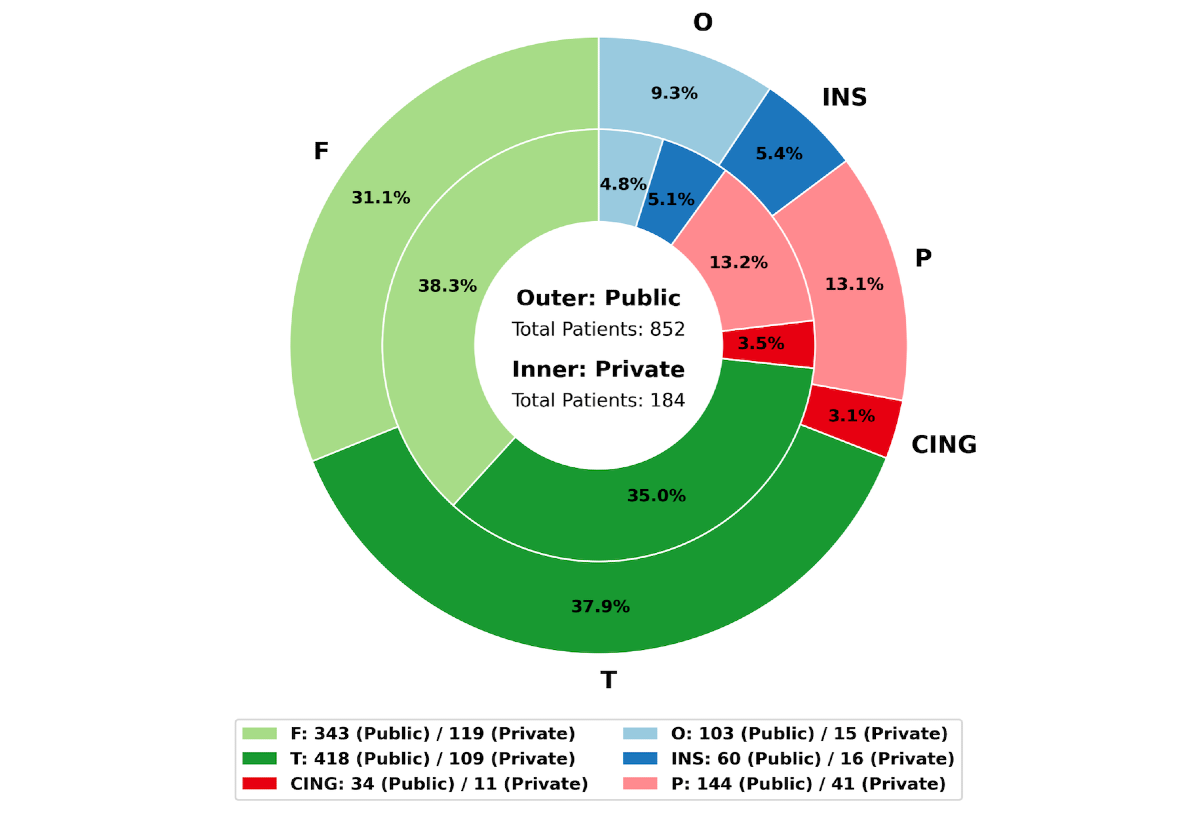
Region distribution for publicly and privately sourced data cohorts. CING: cingulate cortex; F: frontal lobe; INS: insular cortex; O: occipital lobe; P: parietal lobe; T: temporal lobe.

As illustrated in Figure 2, the distributions of EZs in publicly sourced and privately sourced cohorts demonstrate remarkable similarity. In the publicly sourced cohort, the temporal lobe (T) and frontal lobe (F) accounted for the most significant proportions of EZ-semiology cases, followed by the parietal lobe (P) and occipital lobe (O), with the insular cortex (INS) and cingulate cortex (CING) comprising the most diminutive proportions. The privately sourced cohort exhibited a similar distribution, with a minor variation where the frontal lobe slightly surpassed the temporal lobe in the number of semiology-EZ cases.

The lobe distribution from both privately and publicly sourced data cohorts highlights that the temporal and frontal lobes were the most frequently and commonly identified lobes with EZs, not only in the context of research studies but also in real-life clinical practice. Cases with EZs from the parietal and occipital lobes were comparatively less frequent. At the same time, those involving the insular and cingulate cortex remained rare and consistent for privately and publicly sourced data cohorts.

### Patient Demographics

The publicly sourced data cohort comprised 852 semiology-EZ pairs from 852 patients. It included 320 female patients, 404 male patients, and 128 patients with undisclosed gender. Of all 852 patients, 134 were right-handed, 22 were left-handed, 3 were ambidextrous, and 706 had unspecified handedness. The age range of the patients spanned from newborn to 77 years, with 310 individuals younger than 18 years and 335 individuals 18 years old or older. The average age was 23.66 (SD 17.16) years.

The FEMH data cohort consisted of 184 semiology-EZ pairs. This group included 44 female and 46 male patients, and gender information was unavailable for the others. The patients’ ages ranged from newborn to 87 years. Among them, 37 were younger than 18 years, and 50 were 18 years old or older. The average age was 28.67 (SD 18.05) years.

### Response Generation With ChatGPT

For this study, we selected ChatGPT-4 as the primary LLM example due to its superior performance compared with ChatGPT-3.5. The detailed results of ChatGPT-3.5 are included in Multimedia Appendix 1 for reference. To evaluate the performance of ChatGPT-4, we used the “gpt-4-turbo” application programming interface and implemented 2 distinct prompt configurations: zero-shot prompting (ZSP) [21] and few-shot prompting (FSP) [22].

In the ZSP configurations, ChatGPT received no prior examples nor guidance and relied solely on its internal knowledge to make predictions. In contrast, the FSP configurations incorporated 3 randomly selected semiology-EZ pair examples as input, guiding ChatGPT’s predictions to align more closely with the ground truth. These 3 input examples were the same prompts across all test samples to ensure consistency during evaluation.

All semiology information was formatted into a fixed sentence structure to standardize query sentence design and ensure robust predictions. When patient-specific details were available, the input combined these details with the semiology into a single sentence, such as: “A [handedness] [gender] patient, aged [age], presented with semiology: [semiology].” In cases where patient-specific information was unavailable, the input was simplified to: “The patient presented with semiology: [semiology].” ChatGPT was then tasked with predicting the most likely EZs based solely on the provided semiology, with its output restricted to specifying the EZ location without any accompanying explanation. Examples of query formats and configurations used in this study are provided in Table 1.

**Table 1:** Examples of questions and responses generated by ChatGPT with different prompt configurations.

Action	Zero-shot prompting	Few-shot prompting
User input	One sentence including the patient’s semiology and demographic information	One sentence including the patient’s semiology and demographic information
Prompt	None	Case 1: a right-hand male patient, aged 24, presented with semiology: a slightly painful sensation of tightness in the right shoulder and the region of the right sternocleidomastoid muscle; EZ^a^: frontal lobe Case 2: a patient presented with semiology: gastric aura and a warm feeling in the chest, rising to her face, facial flushing, followed by oroalimentary automatism; EZ: temporal lobe Case 3: a patient presented with semiology: aura, loss of contact, motor; EZ: occipital lobe
Query	Based on the user’s semiology, list the most likely epileptogenic zones (EZ) in descending order of likelihood. The epileptogenic zones include the frontal lobe, temporal lobe, parietal lobe, occipital lobe, cingulate, and insular cortex. Provide the answer in this format: ‘EZ1, EZ2, ...’. If there is only one likely EZ, list only that one. Do not include any explanations.	Based on the user’s semiology, list the most likely epileptogenic zones (EZ) in descending order of likelihood. The epileptogenic zones include the frontal lobe, temporal lobe, parietal lobe, occipital lobe, cingulate, and insular cortex. Provide the answer in this format: ‘EZ1, EZ2, ...’. If there is only one likely EZ, list only that one. Do not include any explanations.
Description	A right-handed male patient, aged 38, presented with semiology: speech arrest	A right-handed male patient, aged 38, presented with semiology: speech arrest
Response	Frontal lobe	Frontal lobe, temporal lobe

^a^EZ: epileptogenic zone.

### Evaluation of Seizure Semiology Interpretation From Epileptologists

To evaluate ChatGPT’s clinical value for interpreting seizure semiology, we compared its performance with that of a panel of board-certified epileptologists [23] using the voluntary seizure semiology survey [24]. The research team initially tested the survey’s usability and technical functionality before its public debut. After optimization and improvement, the final version was deployed for response collection.

This survey was conducted using an open online survey platform named Zoho Survey, which is a free online tool that facilitates automated survey creation and response collection. It was structured into multiple sections to ensure clarity and ease of participation. The first page provided participants with essential instructions, including the total number of questions, the estimated time required, guidelines on completing each query, and instructions on how to save progress. An example was also included for better instruction. The second page collected bibliographic details of participants, which were strictly protected and used solely for research purposes. From the third page onward, participants had to complete 100 compulsory multiple-choice questions, each presenting a unique seizure semiology description. Participants were asked to determine the most likely EZ from 6 options: frontal lobe, temporal lobe, parietal lobe, occipital lobe, cingulate cortex, and insular cortex. There was an additional “Other” comment box for alternative responses. Each page displayed a single patient case, and participants could proceed to the next question by clicking the “Next” button. If participants wished to revise a previous response, they could navigate back using the “Previous” button. The last page demonstrated appreciation for their participation.

To track and analyze the participants’ performance, the Zoho platform automatically recorded visitors’ IP addresses to determine the total number of unique participants and tracked survey progress, allowing researchers to identify whether a participant only completed the bibliographic section or proceeded to answer the questions. To prevent data loss, participants were instructed to press the “Save and Continue Later” button (available from the second page onward) if they needed to take a break. Additionally, surveys submitted with atypical timestamps were flagged for further review to ensure data integrity and reliability.

To minimize selection bias regarding region or semiology, we randomly selected a subset of 100 semiology records covering all 6 general brain regions from our self-compiled database. The final subset used in the survey met the following criteria: (1) Selected semiology records contained comprehensive and explicit descriptions of seizure symptoms, (2) the distribution of EZs spanned all 6 general regions rather than focusing on 1 region, and (3) the records were chosen to capture the broadest possible range of seizure symptoms.

Epileptologists were recruited through the National Association of Epilepsy Center and the American Epilepsy Society, with over 70 survey invitation emails and survey links distributed worldwide. Responses were collected between January 2024 and July 2024. Of the 8 epileptologists who participated, 6 completed the survey in full, while 2 provided partial responses. All participating epileptologists were employed at different epilepsy centers during the survey, with clinical experience ranging from 7 years to 35 years. Their affiliations spanned the western, central, and eastern regions of the United States and Germany, ensuring geographic diversity.

For comparative analysis, 5 epileptologists were selected from the 6 who completed the survey, given that 1 epileptologist was primarily employed as a neuro-oncologist. These selected specialists served as a benchmark to evaluate ChatGPT’s accuracy at semiology-based EZ localization. To ensure consistency, the input format presented to the epileptologists mirrored the structure of the queries used for ChatGPT. Epileptologists identified the most likely EZ locations based on the provided semiology. Comparative examples of outputs from ChatGPT and the selected epileptologists are presented in Table 2.

**Table 2:** Performance comparison of zero-shot prompting (ZSP) semiology interpretation between GPT-4 and epileptologists 1-5 (E1-E5).

Number	Semiology interpretation query	Ground truth	GPT-4	E1	E2	E3	E4	E5
1	The patient is a 14-year-old right-hand male. His reported semiology is speech arrest and lower speech intensity. According to the patient’s reported semiology, what are the most likely general lobes of the brain where the epileptogenic zone is located? (multiple choice question)	INS^a^	F^b^, INS	F	F	F, T^c^	F, INS	F, T
2	The patient, a right-handed 67-year-old female, began experiencing seizures at the age of 66. During 2 of the 9 recorded seizures, she exhibited symptoms of drooling, spitting, and coughing. According to the patient’s reported semiology, what are the most likely general lobes of the brain where the epileptogenic zone is located? (multiple choice question)	P^d^	T, INS	T, INS	T	F, INS	F, INS	F
3	The patient is a 43-year-old left-hand male. His age at seizure onset was 6 years old. His reported semiology is a dreamy state, loss of consciousness, oroalimentary and gestural automatisms, nose wiping, and right arm dystonic posturing. According to the patient’s reported semiology, what are the most likely general lobes of the brain where the epileptogenic zone is located? (multiple choice question)	T	T	T	T	F, T	T	T

^a^INS: insular cortex.

^b^F: frontal lobe.

^c^T: temporal lobe.

^d^P: parietal lobe.

### Statistical Analysis

The inference of EZ location was determined using the 6-lobe classification criteria. To evaluate the responses from ChatGPT and epileptologists, we used 3 metrics: regional sensitivity (RSens), weighted sensitivity (WSens), and net positive inference rate (NPIR).

Sensitivity is a widely used metric in classification problems [25]. The study calculated the overall sensitivity and evaluated the sensitivity for each EZ region. Specifically, RSens measured the accuracy of ChatGPT or epileptologists in identifying the correct region and was defined as follows:




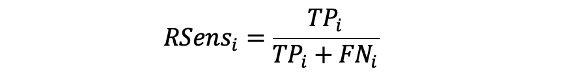




where *i* denotes the index corresponding to 6 general regions, *Rsens_i_* denotes the sensitivity value for region *i*, *TP_i_* (true positive) denotes the number of EZs that were part of the ground truth and correctly identified for a data set, and *FN_i_* (false negative) denotes the number of ground-truth EZs that were not identified for a data set.

For example, consider a data set of 100 semiology-EZ samples. The region mapping is as follows: 0-Cingulate (CING), 1-Frontal (F), 2-Insular (INS), 3-Occipital (O), 4- Parietal (P), and 5-Temporal (T). If 80 cases have the true label F, only 60 are correctly identified as F (*TP*_1_=60), while the remaining 20 are mislabeled as other regions (*FN*_1_=20); the sensitivity for the frontal lobe is calculated as:




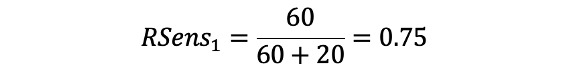




The result means that 75% of the F cases are correctly identified.

Additionally, given the unbalanced distribution of EZs across the 6 general regions, we addressed the class imbalance issue using WSens to provide a more accurate performance assessment. WSens evaluates overall accuracy by considering the RSens of each region and its corresponding weight in the data set, which is calculated as follows:




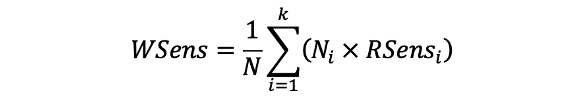




where *k* denotes the total number of regions, *i* denotes the index corresponding to each region, *N* denotes the total number of regions in the data set, and *N_i_* denotes the count of instances for the *i*-th region.

For instance, consider a data set with 130 cases distributed across 6 regions, with the distribution of instances (*N_i_*) and their corresponding *Rsens_i_* as follows: *N*_0_=10, *Rsens*_0_=0.1; *N*_1_=40, *Rsens*_1_=0.7; *N*_2_=5, *Rsens*_2_=0.5; *N*_3_=15, *Rsens*_3_=0.6; *N*_4_=20, *Rsens*_4_=0.4; *N*_5_=40, *Rsens*_5_=0.58. The N is calculated as *N*=10+40+5+15+20+40=130, and WSens is computed as:




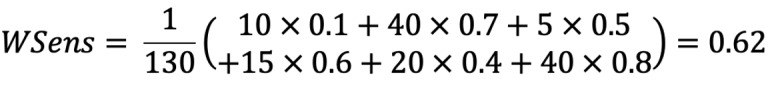




The result indicates an overall accuracy of 62% across 6 regions.

Although sensitivity for an individual region and across 6 regions has been extensively discussed, the performance evaluation for each semiology query—representing an individual patient’s case—has not been addressed. This aspect is particularly significant for clinical practice. The NPIR derived from RSens is introduced to bridge this gap. Although the denominator remains unchanged, the numerator is adjusted to reward correctly inferred regions for a given semiology while penalizing incorrect inference. The NPIR is calculated as follows:




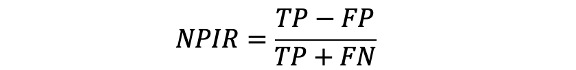




where *TP* denotes the number of EZs that were part of the ground truth and correctly identified for a single semiology query; *FP* (false positive) denotes the number of EZs that were not part of the ground truth but were incorrectly identified as such; and *FN* denotes the number of ground-truth EZs that were not identified. For example, consider a patient’s semiology query with the true labels F and T. If the localization result includes T, P, and O, then *TP*=1 (for T, correctly identified), *FP*=2 (for P and O, incorrectly identified), and *FN*=1 (for F, missed in the prediction). The NPIR is:




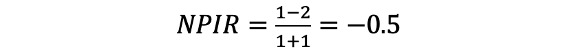




This result indicates that the model provides partially correct localizations and identifies misleading EZ locations.

Regarding value interpretation, an NPIR value reflects the reliability of the inference results for a single semiology query. An NPIR of 1 indicates an utterly correct inference of the EZ location. A value less than 1 suggests the inference is partially incorrect or contains omissions. An NPIR less than 0 indicates that the inference is unreliable and could mislead physicians during preoperative assessments for epilepsy surgery.

In summary, RSens quantifies the ability to accurately identify EZs for specific brain regions and provides insights into a tool’s performance on individual regions in a clinical context. Although RSens values range between 0 and 1, they are better interpreted as percentages to represent the proportion of correctly identified EZs in a given region. Similarly, WSens accounts for class imbalance by weighting regional sensitivities, offering an overall accuracy metric that reflects performance across all regions. Like RSens, WSens should also be interpreted as a percentage for ease of understanding in clinical applications.

By contrast, NPIR assesses the reliability of predictions by penalizing incorrect inferences. Higher NPIR scores indicate more trustworthy results, while negative scores highlight misleading localizations, both of which are critical for clinical practice. However, NPIR is influenced by the number of identified regions provided by ChatGPT or epileptologists. When multiple EZ localizations are made for a single case, NPIR values often cluster around 0 or below 0, reflecting the trade-off between partially correct and incorrect inferences.

More importantly, RSens, WSens, and NPIR offer complementary layers of analysis: RSens evaluates performance at the level of individual EZs, WSens aggregates sensitivity across six EZ regions, and NPIR assesses inference accuracy for each semiology query.

## Results

### Evaluation of Responses From ChatGPT on the Publicly Sourced Cohort

**Figure 3:**
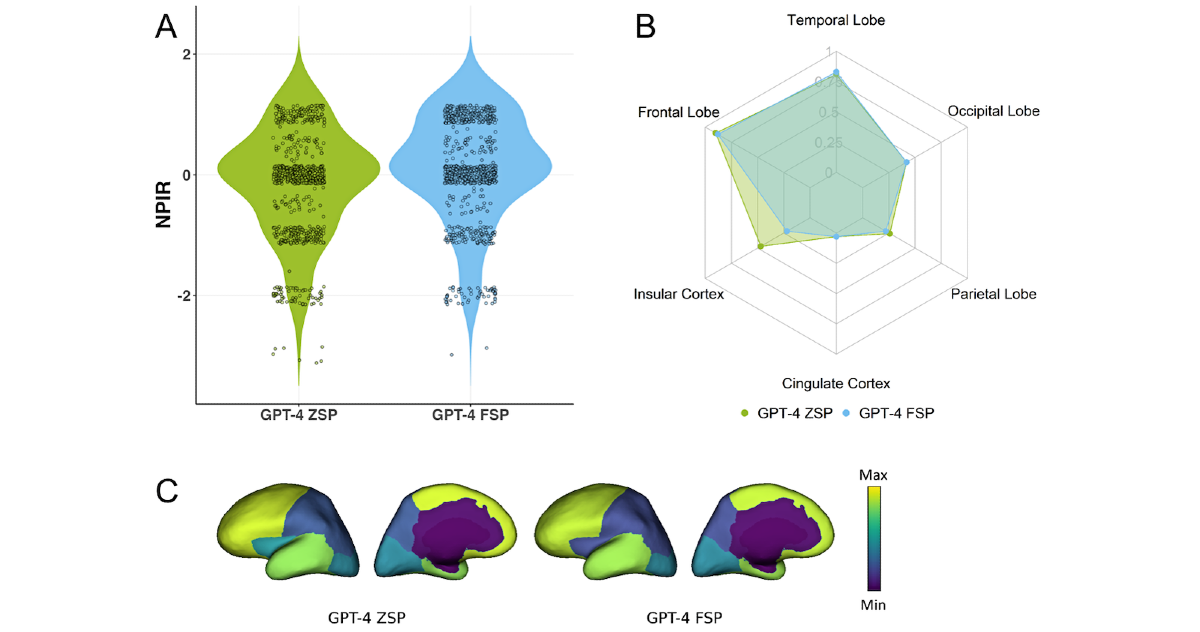
Performance comparison on a publicly sourced data cohort between ZSP and FSP using ChatGPT-4: (A) NPIR distribution; (B) and (C) RSens.

In this section, we evaluated the performance of ChatGPT-4 using ZSP (abbreviated as GPT-4 ZSP) and ChatGPT-4 using FSP (GPT-4 FSP) in interpreting seizure semiology based on the publicly sourced cohort. The evaluation results for ChatGPT-4 are presented in Figure 3, while those for ChatGPT-3.5 are provided in Multimedia Appendix 1.

Figures 3B and 3C present the RSens values for each region. For the frontal lobe, GPT-4 ZSP achieved 0.90, and FSP achieved 0.88. For the temporal lobe, ZSP achieved 0.81, and FSP achieved 0.83. For the occipital lobe, both ZSP and FSP achieved 0.42. For the parietal lobe, ZSP achieved 0.26, and FSP achieved 0.22. For the insular cortex, ZSP achieved 0.47, and FSP achieved 0.22. For the cingulate cortex, both ZSP and FSP achieved 0.03.

These RSens results highlight ChatGPT’s proficiency at interpreting seizure semiology for the frontal and temporal lobes, the most commonly observed regions in the public and private data cohorts. However, performance declined for the occipital lobe, parietal lobe, and insular cortex, which were less frequently represented in the distribution. For the cingulate cortex, the least commonly involved region observed in both data cohorts, performance remained consistently low across both prompting methods.

Regarding WSens, GPT-4 ZSP and GPT-4 FSP demonstrated comparable results, with WSens values of 0.69 and 0.67, respectively. Although these values are not exceptionally high, they should be evaluated compared with epileptologists’ performance to determine the relative accuracy and potential utility of ChatGPT-4 for assisting seizure semiology interpretation.

As shown in Figure 3A, the NPIR values, which reflect the interpretation accuracy for each semiology, indicated that GPT-4 ZSP achieved a mean of –0.21, while GPT-4 FSP demonstrated a slightly higher mean of 0.03. An NPIR close to 0 indicates that ChatGPT-4’s interpretations often include at least one correctly identified region but may also involve one misleading region.

### Evaluation of Responses From ChatGPT With the Privately Sourced Cohort

**Figure 4:**
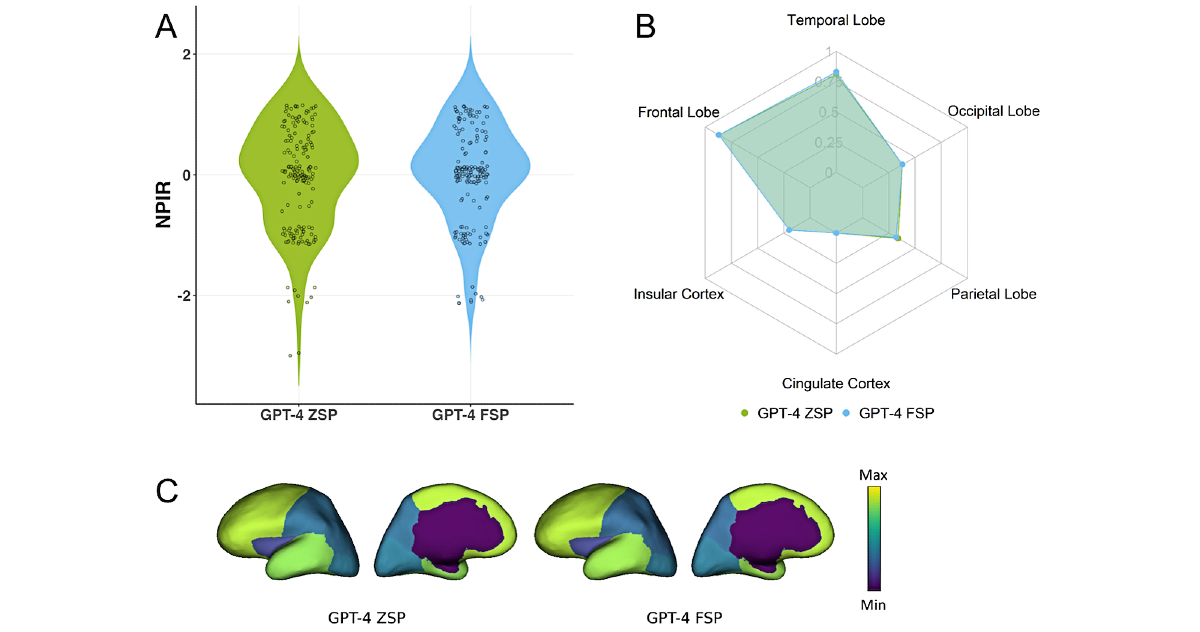
Performance comparison on a privately sourced data cohort between ZSP and FSP using ChatGPT-4: (A) NPIR distribution; (B) and (C) RSens.

Given that all papers used to compile the publicly sourced database are available online, some may have been included in ChatGPT’s training corpus, potentially making the evaluation results less objective and convincing. To address this concern, we used a database from a private source for external validation of ChatGPT’s performance. The evaluation results for this database are presented in Figure 4.

Figures 4B and 4C present the RSens values for each brain region. For the frontal lobe, GPT-4 ZSP and FSP achieved an RSens value of 0.87. For the temporal lobe, ZSP achieved 0.81, while FSP achieved 0.83. For the occipital lobe, both methods achieved 0.38. For the parietal lobe, ZSP achieved 0.34, while FSP achieved 0.32. ZSP and FSP achieved 0.20 for the insular cortex, and for the cingulate cortex, both recorded a value of 0.

Regarding WSens, GPT-4 ZSP achieved a value of 0.73, while FSP slightly outperformed it with 0.74. For NPIR, GPT-4 ZSP achieved a mean of –0.20, while FSP demonstrated a slightly improved mean of –0.12, as shown in Figure 4A.

These evaluation results based on the private data source cohort are consistent with those from the publicly sourced cohort, further confirming ChatGPT’s ability to interpret seizure semiology across different brain regions.

### Comparison of Responses From ChatGPT and Epileptologists

**Figure 5:**
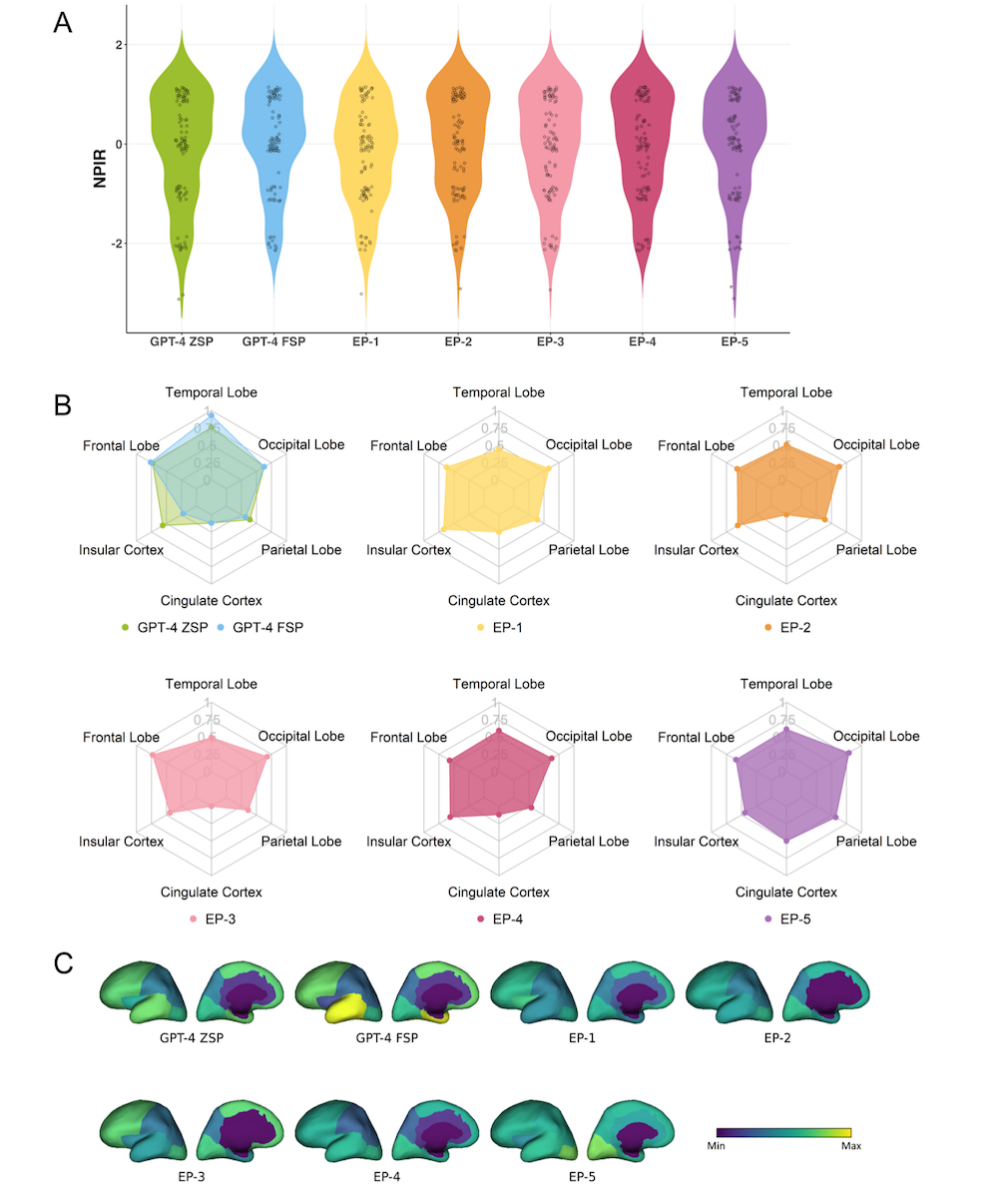
Performance comparison on a 100-question survey between ChatGPT-4 using ZSP and FSP, and five individual epileptologists (EP-1 to EP-5) using ZSP: (A) NPIR distributions; (B) and (C) RSens.

In this section, we compared the performance of ChatGPT with that of 5 board-certified epileptologists (EP-1, EP-2, EP-3, EP-4, EP-5). The comparison results are shown in Figure 5.

When comparing RSens values across regions (Figures 5B and 5C), ChatGPT-4 outperformed epileptologists at interpreting seizure semiology for the frontal (ZSP: 0.73; FSP: 0.76; epileptologists: 0.57-0.73) and temporal lobes (ZSP: 0.76; FSP: 0.93; epileptologists: 0.44-0.61) and demonstrated comparable performance for the parietal lobe (ZSP: 0.39; FSP: 0.32; epileptologists: 0.29-0.57), occipital lobe (ZSP: 0.63; FSP: 0.63; epileptologists: 0.58-0.79), and insular cortex (ZSP: 0.56; FSP: 0.22; epileptologists: 0.44-0.67). However, it underperformed epileptologists at interpreting seizure semiology cases associated with the cingulate cortex (ZSP and FSP: 0.12; epileptologists: 0-0.5). These results indicate that ChatGPT-4 outperforms epileptologists at interpreting seizure semiology for regions more commonly associated with EZ, such as the frontal and temporal lobes. However, its performance declines for less frequently observed regions in public databases (representing research settings) and private databases (reflecting real-world clinical contexts).

In terms of WSens, ChatGPT-4 significantly outperformed the epileptologists. Specifically, GPT-4 ZSP achieved a WSens of 0.61, and GPT-4 FSP achieved a slightly higher WSens of 0.63. In comparison, the WSens of epileptologists ranged from 0.49 (EP-2) to 0.51 (EP-5). These findings highlight that ChatGPT-4 delivers more consistent accuracy across all regions than epileptologists.

For NPIR, as illustrated in Figure 5A, GPT-4 ZSP achieved a mean of –0.14, while GPT-4 FSP achieved a mean of –0.02. Among the epileptologists, EP-5 achieved the highest mean NPIR of –0.08, while EP-2 recorded the lowest mean of –0.13. The performance of both GPT-4 and the epileptologists remained near 0, indicating comparable performance.

### Significant Testing of Responses From ChatGPT and Epileptologists

**Figure 6:**
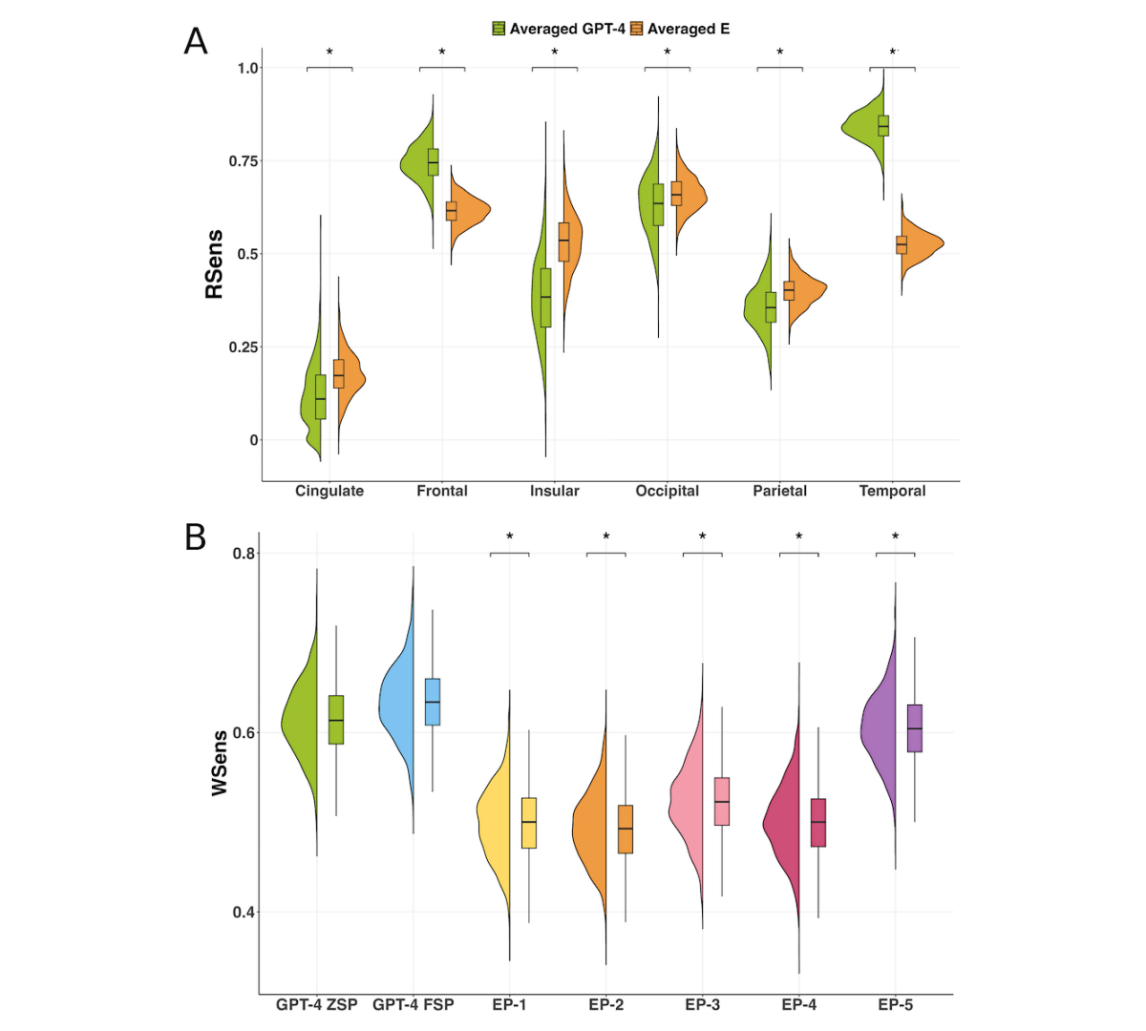
Significance testing comparison of ChatGPT-4 and epileptologists: (A) RSens comparing averaged GPT-4 performance (across ZSP and FSP) with averaged epileptologist performance (E); (B) WSens comparing GPT-4 (ZSP and FSP) with five individual epileptologists (EP-1 to EP-5). *P<.001.

To evaluate whether the differences in RSens performance between ChatGPT and epileptologists were statistically significant, we used data from the survey of 100 semiology cases. A group *t* test was conducted with 1000 bootstrap samples to determine the significance of these differences. The comparison focused on the averaged RSens performance of ChatGPT-4 (using ZSP and FSP) and the averaged performance of 5 epileptologists. As illustrated in Figure 6A, the results indicated significant differences, suggesting that ChatGPT-4 excels at commonly represented EZs like the frontal lobe and temporal lobe but faces challenges with rarer EZs, such as the cingulate cortex.

For WSens, a similar group *t* test with 1000 bootstrap samples was used to assess the overall performance differences between ChatGPT-4 using ZSP, ChatGPT-4 using FSP, and the 5 epileptologists. As shown in Figure 6B, ChatGPT-4 significantly outperformed the group of epileptologists in overall sensitivity. Among the 5 epileptologists, EP-1 and EP-4 did not show a statistically significant difference in performance, whereas the remaining epileptologists demonstrated significant differences from one another, with *P* values <.001.

Interestingly, the years of clinical experience among epileptologists, which ranged from 7 years to 35 years, were not consistently correlated with performance. For instance, EP-1 (20 years of experience) and EP-4 (8 years of experience) demonstrated similar performance levels, while EP-3, the least experienced with 7 years of practice, ranked among the top 2 performers. In contrast, EP-2 (16 years of experience) showed the lowest performance, whereas EP-5, the most experienced with 35 years of practice, achieved the highest performance in the group. These findings suggest that clinical experience alone may not guarantee consistent reliability, highlighting the potential value of supplementary tools, such as ChatGPT, to assist with clinical decision-making.

For NPIR, as shown in the “Comparison of Responses from ChatGPT and Epileptologists” section and Figure 6B, the distributions of NPIR values for ChatGPT-4 ZSP, ChatGPT-4 FSP, and the 5 epileptologists were visually similar. Since we already had data distributions, a direct *t* test was conducted on the 100 survey entries. The results indicated no statistically significant differences in NPIR performance between ChatGPT-4 and the epileptologists.

## Discussion

### Principal Findings

We evaluated ChatGPT’s capability for interpreting seizure semiology to localize the EZ using a publicly sourced cohort with 852 semiology-EZ pairs from peer-reviewed journals, a private data cohort with 184 pairs from FEHM, and a survey with 100 randomly selected semiology queries.

Regarding the analysis of RSens, ChatGPT achieved RSens values of approximately 80% to 90% in the most commonly observed regions, such as the frontal and temporal lobes, in both the public and private databases. However, RSens declined as the regions became less common, including the occipital lobe, parietal lobe, and insular cortex, and dropped significantly for rare regions like the cingulate cortex, where ChatGPT failed to localize correctly in most cases. These findings align with the comparison between ChatGPT and epileptologists in the survey. ChatGPT significantly outperformed epileptologists at identifying EZ locations in more common regions, demonstrated comparable but slightly lower performance in less common regions, and substantially underperformed in rare regions.

The higher performance on common semiology-based EZ localization query and lower performance on less common questions observed in this study is consistent with findings from previous studies assessing ChatGPT’s performance in epilepsy-related inquiries. Specifically, Kim et al [26] assessed the reliability of responses by ChatGPT to 57 commonly asked epilepsy questions, and 2 epileptologists reviewed all responses. The results suggested that these responses were either of “sufficient educational value” or “correct but inadequate” for almost all questions. Wu et al [27] evaluated the performance of ChatGPT on a total of 378 questions related to epilepsy and 5 questions related to emotional support. The statistics indicated that ChatGPT provided “correct and comprehensive” answers to 68.4% of the questions. However, ChatGPT performed poorly when answering “prognostic questions”; only 46.8% of answers were rated as comprehensive. These findings suggest that ChatGPT’s performance is positively correlated with the availability of sufficient data to support its responses.

Although ChatGPT’s performance on RSens varies depending on the EZ localization of seizure semiology, it remains clinically valuable. This is because, in most real-world cases, patient symptoms align with more common seizure types, such as frontal lobe and temporal lobe seizures. These 2 types accounted for 80% of the cases in the private data cohort from the real-life hospital. Furthermore, regarding WSens, ChatGPT achieved over 60% accuracy in both the public and private databases and significantly outperformed the 5 epileptologists, demonstrating its strong generalizability in seizure semiology interpretation.

Given these findings, ChatGPT could serve different roles depending on the clinical setting. In epilepsy centers with rich resources, it may function as a copilot to support epileptologists at improving diagnostic efficiency. In resource-limited epilepsy centers, where access to specialized epilepsy care is scarce, ChatGPT could be particularly valuable for assisting general practitioners or nonspecialist clinicians with preliminary seizure classification and decision-making, potentially improving access to epilepsy care.

Besides those metrics, ChatGPT has the potential to assist further and enhance clinical decision-making in epilepsy centers to achieve optimal postsurgical outcomes since it could make presurgical EZ identifications based on an LLM trained with a large cohort of cases. At the same time, epileptologists could only localize according to their clinical experience, laboratory results, and patients’ self-report.

### Limitations

Although this study offered an important reference on the capability of ChatGPT to interpret the descriptions of seizure semiology to localize EZs, several limitations remain.

First, when inferring the EZ location according to semiology, the identified area is termed the symptomatogenic zone, which is the region responsible for the observed seizure symptoms but may not fully align with the actual EZ, which is a theoretical definition given by Lüders et al [4]; whether removal of the EZ will make the patients seizure-free thus can only be derived after surgical ablation. The findings of this study will result in limited precision when identifying EZs. Additionally, epileptic seizures often involve abnormal activities across multiple brain regions, with specific symptoms arising from the propagation of activity to regions beyond the EZ, which may lead to potential misjudgments.

Second, the database used to train and evaluate ChatGPT in this study could be further expanded and refined. The 2 databases have inadequate data for less common regions, such as the cingulate cortex and insular cortex. Additionally, ChatGPT was trained on the Common Crawl corpus, which encompasses a broad range of general knowledge but lacks a specific focus on medical or epilepsy-related information. This lack of specialized data limits ChatGPT’s ability to generate precise and reliable responses for semiology interpretation and EZ localization.

Third, there may be biases in ChatGPT’s output due to the underlying training cohorts. For instance, the data sets predominantly include cases from Europe, East Asia, and North America, with significantly fewer cases from South America and Africa. Additionally, the data broadly represent patients who can afford epilepsy surgery. As a result, interpretation results might be influenced by this nonrepresentative sampling of patients worldwide.

Last, the limited number of participating epileptologists inherently restricts the sample size for more comprehensive analysis. Insights from a small group of epileptologists may not adequately reflect the broader expertise and perspectives of epileptologists from the global community.

### Future Work

As mentioned in this study, there is an imbalance of semiology data distribution across the 6 lobes, with most cases originating from the frontal and temporal lobes. To mitigate this bias in this study, we incorporated WSens analysis to account for differences in EZ distribution. Additionally, we plan to address this limitation in future studies by collecting more data on less common epilepsy regions and fine-tuning LLMs using epilepsy-specific corpora. Finally, a sequential description of seizure semiology can help map out the seizure propagation pathways from seizure onset zones to other symptomatogenic brain regions. It is important to leverage the sequence information of seizure semiology to provide a detailed characterization of the epileptic brain network.

## Appendix

Multimedia Appendix 1 Performance Comparison between Zero-Shot Prompting (ZSP) and Few-Shot Prompting (FSP) on ChatGPT-3.5, including: (A) Net Positive Inference Rate (NPIR) on the publicly sourced database; (B) Regional sensitivity (RSens) on the publicly sourced database; (C) NPIR on the privately sourced database; (D) RSens on the privately sourced database; (E) NPIR on the 100-question survey; and (F) RSens on the 100-question survey.

## Abbreviations

CING: cingulate cortex
EHR: electronic health record
EP-1: epileptologist 1
EP-2: epileptologist 2
EP-3: epileptologist 3
EP-4: epileptologist 4
EP-5: epileptologist 5
EZ: epileptogenic zone
F: frontal lobe
FEMH: Far Eastern Memorial Hospital
FSP: few-shot prompting
INS: insular cortex
LCN-CortLobes: Laboratory for Computational Neuroimaging Cortical Lobes Dataset
LLM: large language model
NPIR: net positive inference reliability
O: occipital lobe
P: parietal lobe
RSens: regional sensitivity
T: temporal lobe
WSens: weighted sensitivity
ZSP: zero-shot prompting

## Data Availability

The data will be made available upon reasonable request to the corresponding author, FL. 
