# Supplementary figures and images for: Clinical Value of ChatGPT for Epilepsy Presurgical Decision-Making: Systematic Evaluation of Seizure Semiology Interpretation
Source: J Med Internet Res. 2025 May 12;27:e69173. doi: 10.2196/69173 (PMC12107199; doi:10.2196/69173)

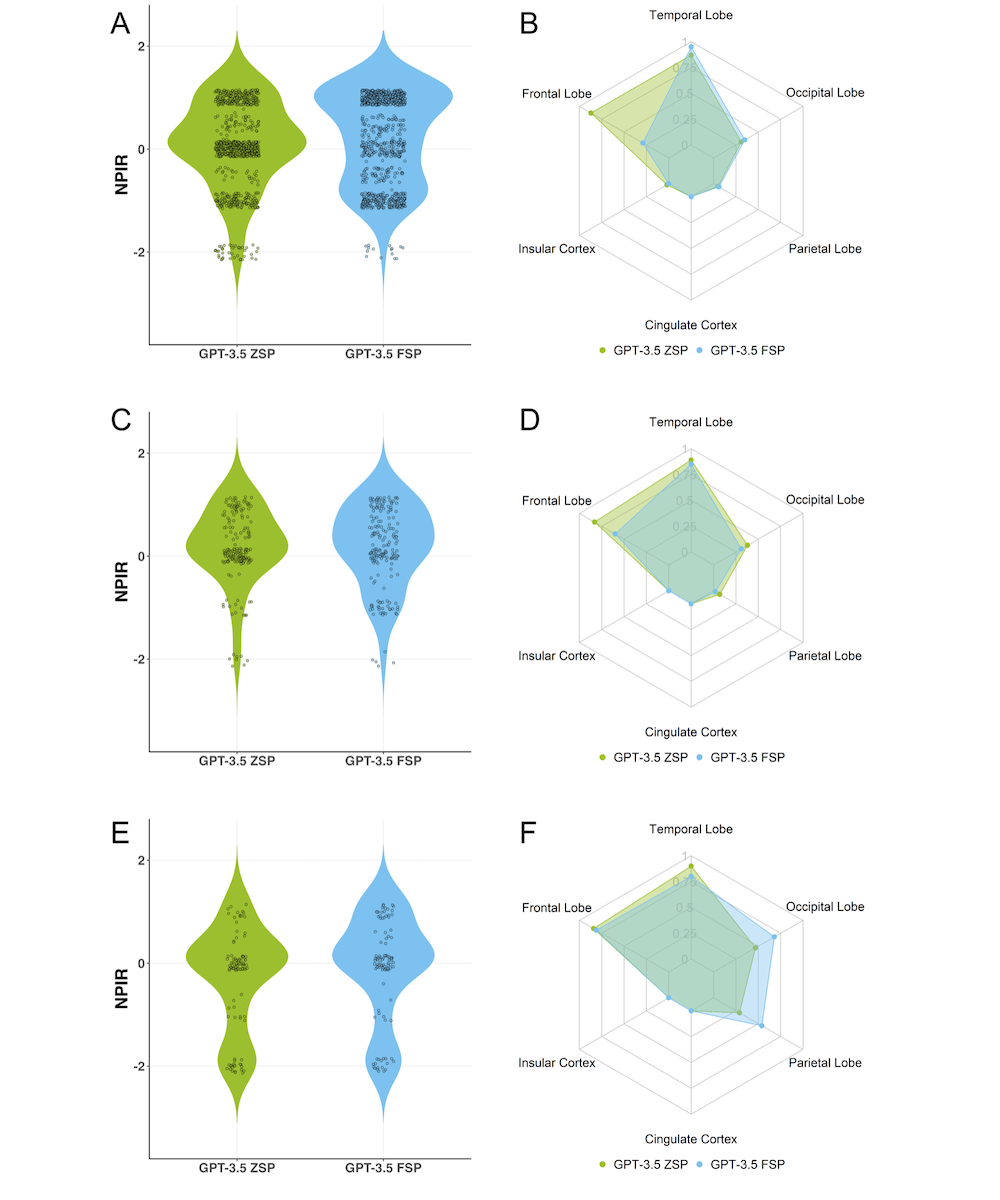

Supplement: Multimedia Appendix 1 [file jmir_v27i1e69173_app1.png]
